# Supplementary material for: Polyethylene Glycol Functionalized Silicon Nanowire Field-Effect Transistor Biosensor for Glucose Detection
Source: Nanomaterials (Basel). 2023 Feb 2;13(3):604. doi: 10.3390/nano13030604 (PMC9919870; doi:10.3390/nano13030604)
Supplement: Supplementary file 1 [file nanomaterials-13-00604-s001.zip › nanomaterials-2071507-supplementary.pdf]

nanomaterials

MDPI

Supplementary Materials

Polyethylene Glycol Functionalized Silicon Nanowire Field-Effect Transistor Biosensor for Glucose Detection

Yan Zhu [1,2,†], Qianhui Wei [2,3,4,†], Qingxi Jin [2,3], Gangrong Li [4], Qingzhu Zhang [5], Han Xiao [1], Tengfei Li [4,*], Feng Wei [2,4] and Yingchun Luo [1,*]

[1] School of Chemical Engineering, Guizhou Minzu University, Guiyang 550025, China
[2] State Key Laboratory of Advanced Materials for Smart Sensing, GRINM Group Co., Ltd., Beijing 100088, China
[3] GRIMAT Engineering Institute Co., Ltd., Beijing 101407, China
[4] GRINM (Guangdong) Institute for Advanced Materials and Technology, Foshan 528051, China
[5] Advanced Integrated Circuits R&D Center, Institute of Microelectronic of the Chinese Academy of Sciences, Beijing 100029, China
* Correspondence: ltf@grinm.com (T.L.); 05111583@gzmu.edu.cn (Y.L.)
† These authors contribute equally to this work.

Section S1 Preparation of glucose solutions and other solutions

Preparation concentration of 0.01 mol/L phosphate buffer (1×PBS): 0.3549 g Na_2HPO_4 and 0.2999 g NaH_2PO_4 were weighed and dissolved in two 250 mL beakers with ionized water, and then appropriate amounts of NaH_2PO_4 solution and Na_2HPO_4 solution were removed and adjusted to 7.4 by pH meter to obtain 0.01 mol/L phosphate buffer. The solutions prepared above were stored in a cool place for subsequent testing.

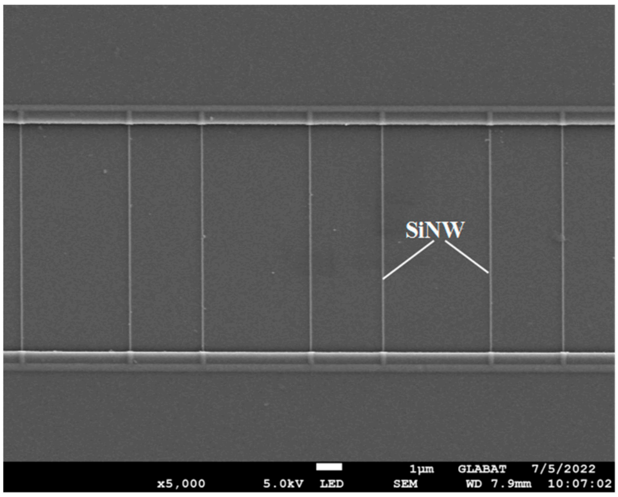

Figure S1. SEM image of the SiNW area.

Section S2 Surface modification examined by AFM imaging

In this study, in order to verify the device surface modification process and GOD immobilization effect, since the surface of the SiNW-FET biosensor is uneven, the silicon wafer is modified by the surface modification steps SiNW-FET biosensor in the text, and the surface modification of silicon wafer is characterized by atomic force microscopy imaging technology. As shown in Figure S2, Figure S2 (a) shows the surface of the bare silicon wafer without modification, which is relatively clean and flat with a roughness of 0.162 nm. After soaking with APTMS:silane-PEG = 2:1 ethanol solution for 30 min, irregular agglomeration can be clearly observed on the surface. The roughness increased from 0.162 nm to 0.548 nm, indicating that silane was successfully immobilized on the wafer surface (Figure S2 (b)). After incubation with GA solution for 1h, the aldehyde group in

GA binds to the terminal amine group on the silicon wafer surface, and the roughness increases to 1.70 nm, indicating that the linking molecule GA has been successfully fixed on the silicon wafer surface (Figure S2 (c)). After incubation with 1.0 mg/mL GOD solution, GOD bound to the end aldehyde group on the wafer surface, and the roughness increased to 1.93 nm, indicating that the recognition probe molecule GOD had been successfully immobilized on the wafer surface (Figure S2 (d)).

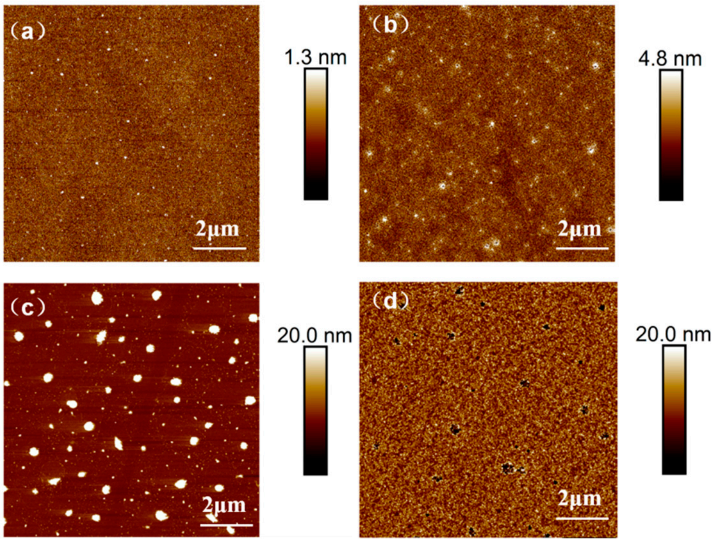

Figure S2. AFM images for the surface modification procedures. (a) a bare SiO_2/Si surface. (b) an APTMS and Silane-PEG co-modified SiO_2/Si area. (c) GA-modified SiO_2/Si area. (d) a zone covered by GOD immobilized on the APTMS modified SiO_2/Si surface.

Section S3 Surface Modification Examined by electrical characterizations

The immobilization of GOD on the SiNW-FET surface was examined by comparing the transfer curves (I_{DS}-V_G) of an SiNW-FET device before and after the modification of GOD. Shown in Figure S3 is an upshift of the transfer curve of a GOD: PEG /SiNW-FET (after modifying GOD, red curve) relative to that of an APTMS: PEG /SiNW-FET (before modifying GOD, black curve). The upshift of the transfer curve (from black to red) was caused by the gating effect when modified with negatively charged GOD in PBS solution to enhance the channel current of p-type SiNW-FET, demonstrating the successful modification of GOD on the SiNW-FET surface.

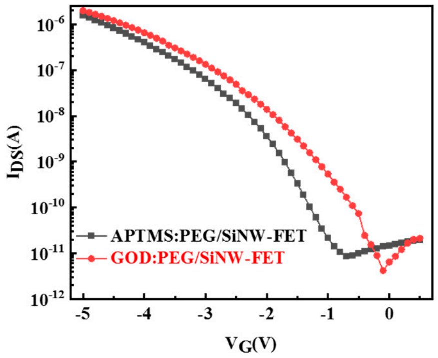

Figure S3. Transfer curve evolution of the SiNW-FET showing the GOD immobilization.

Section S4 Surface Modification Examined by XPS characterizations

In order to confirm the successful immobilization of GOD, a 1x1 cm piece of the silicon wafer was modified in tandem with the actual device and characterized using X-ray photoelectron spectroscopy (XPS). Figure S4 shows the XPS spectra and nitrogen peak allocation of the amine group functionalized surface and GOD functionalized surface. XPS analysis clearly demonstrated the successful immobilization of the glucose oxidase. A new nitrogen (N1s) peak appears in Figure 4S(b), which can be attributed to the required C=N–C bond, as well as the characteristic amide bond (NHC=O) in glucose oxidase.

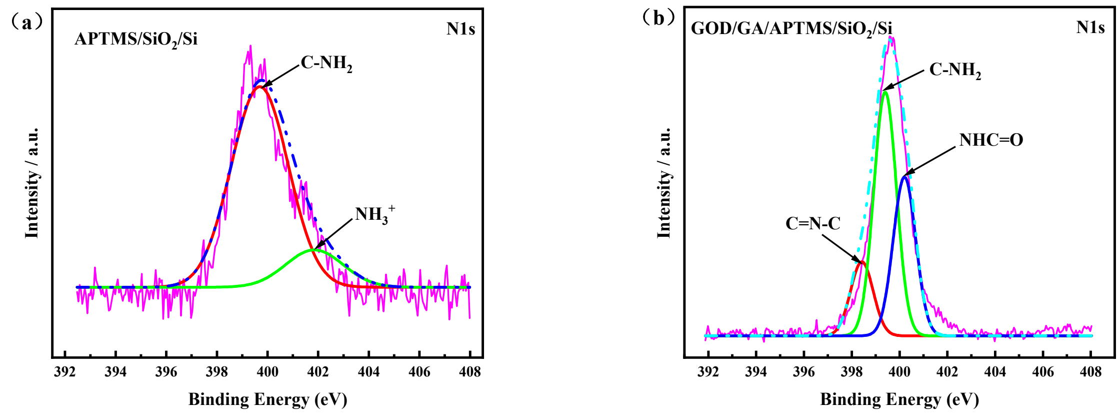

Figure S4. XPS spectra and nitrogen peak assignment for amine-functionalized surface (a) and (b) GOD-functionalized surface. Peak assignment performed according to the literature[1-3].

Section S5 Analysis of why an APTMS/PEG ratio of 2:1 produces the best results

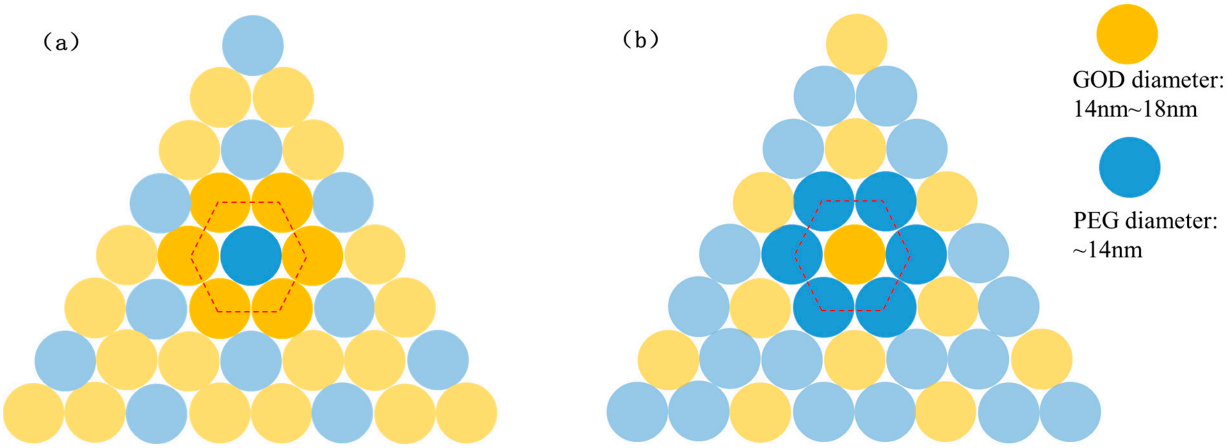

Figure S5. (a) Model assumptions for the modification of APTMS/PEG at a concentration ratio of 2:1. (b) Model assumptions for the modification of APTMS/PEG at a concentration ratio of 1:2.

References

[1] Shircliff, R. A.; Martin, I. T.; Pankow, J. W.; Fennell, J.; Stradins, P.; Ghirardi, M.L.; Cowley, S. W.; Branz, H. M. High-Resolution X-Ray Photoelectron Spectroscopy of Mixed Silane Monolayers for DNA Attachment. *ACS Appl. Mater. Interfaces*. 2011, 3, 3285–3292.

[2] Shircliff, R. A.; Stradins, P.; Moutinho, H.; Fennell, J.; Ghirardi, M. L.; Cowley, S.W.; Branz, H. M.; Martin, I. T. Angle-Resolved XPS Analysis and Characterization of Monolayer and Multilayer Silane Films for DNA Coupling to Silica. *Langmuir*. 2013, 29, 4057–4067.

[3] Meir, R.; Zverzhinetsky, M.; Harpak, N.; Borberg, E.; Burstein, L.; Zeiri, O.; Krivitsky, V.; Patolsky, F. Direct detection of uranyl in urine by dissociation from aptamer-modified nanosensor arrays. *Anal. Chem*. 2020, Anal. Chem. 2020, 92, 12528–12537.
